# The place of health in the EU-CELAC interregional cooperation from 2005 to 2023: a historical, empirical and prospective analysis

**DOI:** 10.1186/s12992-024-01059-3

**Published:** 2024-08-01

**Authors:** Carolina Salgado

**Affiliations:** Pontifical Catholic University of Rio de Janeiro (PUC-RJ), Rio de Janeiro, RJ Brazil

**Keywords:** EU-CELAC cooperation, Health, Change, Economy-driven agenda

## Abstract

**Background:**

Much has been said by actors from different fields and perspectives about the manifold changes in world affairs triggered by the COVID-19 pandemic. In this context, it is to be expected that there will be impacts on long-standing partnerships such as the one between the European Union and the Community of Latin American and Caribbean Countries. However, few studies have demonstrated these impacts, either empirically, by uncovering their specificities or from a historical perspective, to allow for a reasonable methodological comparison of the patterns used to define the partnership and that have changed or have been affected in some way by the pandemic.

**Results:**

Through an in-depth qualitative assessment of primary and secondary sources, this article contributes to this research gap. It analyzes the patterns and changes or impacts in light of two strands of behavior that can make sense of EU-CELAC health cooperation—revisionist or reformist. The findings show an economy-driven health agenda as a new pattern of cooperation, which derives from EU reformist behavior after the pandemic.

**Conclusions:**

The EU power to enforce its priorities in the context of health cooperation with CELAC is the main factor that will define how (and not just which) competing interests and capacities will be accommodated. The relevance of the study to the fields of global governance for health, interregional health cooperation and EU foreign policy is threefold. It shows us i.how two more international regimes are easily intertwined with health—trade and intellectual property—with the potential to deepen asymmetries and divergences even between long-standing strategic partners; ii.contrary to the idea that reformist behaviors are only adopted by actors who are dissatisfied with the status quo, the study shows us that the reformist actor can also be the one who has more material power and influence and who nevertheless challenges the success of cooperation in the name of new priorities and the means to achieve them; and iii.how the EU will find it difficult to operationalize its new priorities internally, among states and private actors, and with those of CELAC, given the history of intense disputes over health-related economic aspects.

## Introduction

Much has been said by actors from different fields and perspectives about the manifold changes in world affairs triggered by the COVID-19 pandemic. Although it was not the first nor the deadliest pandemic in contemporary epidemiological history, it has reinforced, during and after its eclosion (January-March 2020 to May 2023), some disturbing truths underpinning those claims about changes, such as capitalism having no resilience, nationalism comes before cooperation, crises happen simultaneously in cascade effect and, unfortunately, in a number of cases, money matters more than lives.

In this context, it is to be expected that there will be impacts on long-standing partnerships such as the one between the European Union (EU) and the Community of Latin American and Caribbean Countries (CELAC), which was initiated even before the formalization of CELAC occurred at the Summit of the Unity of Latin America and the Caribbean, in the Riviera Maya (Mexico), in 2010. In fact, experts agree that 1999 was a landmark, with the institutionalization of EU-LAC Strategic Partnership, and that “the creation of CELAC in 2010 brought an opportunity for a more structured EU-LAC dialogue, which became organized into EU-CELAC Summits and Action Plans” [39]:IX).

As the examples provided in this research demonstrate with respect to the health area, it is important to note that the EU-CELAC partnership builds on normative and ideational sharing of values and principles historically consolidated, which drives their respective interests and means regarding policies´ implementation. This is why the partnership has been considered here since before CELAC was formalized, and this understanding is supported by publications released by the European Commission and other studies on different areas [5, 51]. It is considered that we cannot grasp recent developments of specific cooperative engagements without knowing how they came into being, especially when the bi-regional relations have a long road.

Zooming a bit in this road, although the EU-CELAC partnership is multifaceted – involving areas such as the strengthening of human rights and democracy; cooperative ties in health, science and technology; support of regionalism and regional spaces for debate and joint actions – it is clear that trade links remain at the center. EU-CELAC experts explain that “various interregional association agreements, economic partnership agreements, multiparty trade agreements and bilateral framework agreements are components of this relationship” [39]:VII), reinforcing each other as trade priorities despite recent changes in the global trade landscape, especially with regard to China's role in CELAC. As for researches, it is common to find compilations [57] and comparative studies [56, 61] involving EU-CELAC manifold agendas, mainly due to the creation of the *EU-LAC Foundation* in 2010 as a tool of the partnership that feeds into the intergovernmental dialogue.

In contrast, few studies have demonstrated impacts of systemic changes (such as the latest pandemic) on EU-CELAC partnership, either empirically, by uncovering their specificities, or from a historical perspective, to allow for a reasonable methodological comparison of the patterns used to define the partnership and that have changed or have been affected in some way by these changes. This article contributes to this research gap through a thorough analysis of empirical documents produced within the EU-CELAC health cooperation over time. In doing so, it also contributes to the literature on EU regional health cooperation, emphasizing the specificities of this case that can be mobilized, in further research, in relation to some of the EU’s other regional health partnerships.^1^ I give a brief overview of this literature and then present the methods of analyzes, explaining why and how they were employed through the study.

The literature on EU regional health cooperation [35, 52, 55, 62], like other regional organizations, draws on the concept of ‘global health governance’ which, for systemic reasons that have to do with globalization [54], emerged simultaneously with the social determinants of health, an approach systematized by the WHO´s Commission on Social Determinants of Health in 2008 [3]. The approach and the related concept indicate that, since health issues are inevitably transnational, cooperation must incorporate a whole array of stakeholders. In this sense, regional organizations act as a bridge between global initiatives and national policy implementation. In the case of the EU, although it has a specific body for health policy within the Commission, which is the DG for Health and Consumers (DG SANCO), according to the Lisbon Treaty its mandate is to act as a complement to national policy-making. In order to do this, the EU establishes strategies^2^ aiming at improving coherence of policy recommendations, “aligning member states on a similar value system for health improvement (…) and reinforce the regional institution´s role as a global actor in health governance” [55]:2–3). Such a role is based on a horizontal integration of public health, considered as a prime objective in all sectors of policy-making [53]. Together with DG SANCO, other EU agencies^3^ are important partners within a wider network that includes a WHO EURO, a regional office of the WHO based in Copenhagen and, very important, the European Public Health Alliance (EPHA), a civil society organization for health cooperation.

## Methods

The article offers a systematization of the patterns of EU-CELAC cooperation in health and their multilateral engagement from a historical perspective, from 2005 until the present, to empirically understand how and what has changed in such patterns since the COVID-19 pandemic. The systematization aims to analyze these changes in light of two strands of behavior that can make sense of EU-CELAC health cooperation – revisionist, meaning "reviewing previous disagreements", or reformist, meaning "setting new priorities". I employ the inductive method of testing the hypothesis stated below through the qualitative content analysis of thirty primary sources available online and listed in the linked references, in addition to secondary literature that dialog directly with these sources. The inductive analysis has the main purpose of uncovering causal mechanisms and interactions effects underlying EU-CELAC health cooperation over time. I aim to understand precisely the logic behind the widespread assumption that the COVID-19 pandemic has triggered manifold changes in world affairs by taking the idea of change seriously. For doing that, the EU-CELAC health cooperation is a paradigmatic case-study because it allows us for a reasonable methodological comparison of the patterns used to define the partnership and that have changed or have been affected in some way by the pandemic.

The subsidiary hypothesis is the following: revisionist behavior is a common pattern among actors who are constantly engaged in interregional relations marked by great asymmetry of power, as is the case with CELAC and the EU in their history of cooperation. This behavior reflects the fact that diffusing the ideas and interests that make up common projects is far from simple [48, 49]. Political coordination around the elements that define the object, instruments and mechanisms of cooperation is permeated by disputes informed by the different identities and worldviews of decision-makers and stakeholders [31, 64]. As a result, we can expect a variety of mechanisms and outcomes for each project that, if qualitatively assessed, reveal relevant aspects about the disputes themselves and, therefore, about the politics of cooperation [36, 37]. In contrast, reformist behavior denotes a significant change in the pattern of cooperation because it raises new priorities that reflect an internal review of foreign policy direction and that may not have been properly negotiated beforehand with counterparties. To the extent that this behavior reforms the cooperation space itself, impacting values and approaches that often happen to facilitate dialog, the reformist actor also challenges the success of the partnership in the name of his priorities and means to achieve them on a given international agenda.

By means of in-depth qualitative assessment of primary sources available online about the projects that paved the EU-CELAC interregional cooperation in health, with further mobilization of secondary literature directly related to these sources, I could identify patterns related to issue areas, outputs, practices and values that are found in all of them until the COVID-19 pandemic and that characterize a rationale of development through health, with a focus on social dimensions. Then, I put these sources in perspective of the EU-CELAC multilateral engagement by mapping the behavior of both partners within the Oslo Group^4^ through their support of the resolutions approved in the United Nations General Assembly (UNGA) over the period of the research, 2008–2020. Such endeavors are in section one, the findings of which indicate that biregional projects did reflect their foreign policy goals within the global governance for health, despite possible disagreements on specific targets, perceptions and methods of interaction. In the second section, I turn to the period after the pandemic to assess what and how changes may have taken place. In sections one and two, I present the respective *results* in order to give a clearer understanding of which and how the referred primary sources were analyzed.

### Preliminary discussion and research goal

By the end of 2021, in a political response to the pandemic, the European Commission set out the Global Gateway strategy, which was followed by the EU Global Health Strategy, launched in November 2022. My claim is that when one looks superficially at health strategy´s three policy priorities, there is not much difference regarding their previous health projects and the resolutions mostly supported by the EU and CELAC countries at UNGA in terms of language, of the way ideas are presented and discourses are written. However, going deeper in the primary sources and carefully analyzing priorities, guiding principles and lines of action of the EU Global Health Strategy, in addition to following news, press releases and communications available mainly in the website of DG International Partnerships, the research indicates that the kind of changes in EU-CELAC interregional cooperation in health, this time, reflects the dominance of EU interests. We can see that the main pattern of interregional cooperation moved from *development through health*, as it was in the previous projects embracing social cohesion, drug policies and policy-oriented health research, to the current *economy-driven health development*—a movement clearly propelled by an EU reformist behavior by means of elevating health technologies and manufacturing as priorities for cooperation with CELAC after the pandemic.

Notwithstanding the fact that such a move was propelled by the EU as observed from its sources that directly address health cooperation with CELAC after the pandemic, one must recognize that, by looking from CELAC, several actions and choices made throughout the pandemic might have influenced the adoption of EU´s changing behavior as displayed in its cooperation strategy with CELAC. It is worth highlighting some examples of CELAC´s initiatives in this regard, of which the *CELAC Plan on Health Self-Sufficiency* [12] discussed within the following sections is the main one. Approved by the XXI Summit of Foreign Ministers of CELAC, held on July 24, 2021 in Mexico, this Plan arises with the idea that Latin America and the Caribbean becomes an actor in the development and production of new vaccines, within the framework of a concerted regional health strategy. There are evidences to support this idea in the large number of technical meetings CELAC has promoted^5^ in order to strength its institutional capacity facing the pandemic – linked to emergencies, preparedness and monitoring, in addition to access and production of medicines, vaccines and strategic supplies.

Moreover, another example seems critical in terms of potentially influence upon the EU: the distribution of respirators, syringes and needles, masks and diagnostic kits donated by China through CELAC. China's cooperation with the region has had to do with the production of vaccines and medicines and the transfer of technologies and sale of pharmaceutical supplies in the region. Therefore, this background allows us to say that: (i) the EU's cooperation with CELAC after the pandemic occurs in a geopolitical context where, on the one hand, China has made significant progress in cooperation with most Latin American countries and, on the other hand, the United States and Europe have lost hegemonic power in the CELAC countries; (ii) the EU´s interests reflected in its renewed strategy are embedded in such geopolitical context, therefore, in some sense reacting to CELAC´s initiatives that have emerged during the pandemic. In this way, the research goal falls upon the EU because changes in patterns used to define the partnership were openly triggered by the EU´s renewed cooperation strategy with CELAC. This fact reinforces the second point made in the abstract, regards the relevance of this study, which I retake here: contrary to the idea that reformist behaviors are only adopted by actors who are dissatisfied with the status quo, the study shows us that the reformist actor – in this case, the EU – can also be the one who has more material power and historical influence, and who nevertheless challenges the success of cooperation in the name of new priorities and the means to achieve them.

To advance the goal of the research—which is to understand how and what has changed in patterns used to define the partnership and which have changed or have been affected in some way by the COVID-19 pandemic—I systematize the analysis in terms of revisionist or reformist behavior. Although the initiatives are yet to be implemented and, therefore, we cannot empirically evaluate their outcomes and reactions, the in-depth qualitative research of several primary sources in addition to the historical path of the politics of cooperation in health between the EU and CELAC within a period of eighteen years in different settings allows me to say that we are witnessing a reformist behavior on the EU side. The EU has redirected its foreign policy with the Global Gateway and a number of instruments, such as Team Europe, and a significant amount of funding for different issue areas and regions. *Economy-driven health development* means that the EU has mobilized its economic power to promote health priorities whose means of implementation are constitutive parts of the main divergences in biregional relations with Latin America and the Caribbean, as will be seen below.

In the third section, therefore, I discuss the results. I take as a point of reference how historical differences in approaches to health between the two regions have been negotiated, with a focus on the issue of pharmaceutical manufacturing and health technologies. Its aim is to support a plausible interpretation of the impact that changing EU behavior and priorities may have on the EU-CELAC partnership in the field of health within the framework of the EU Global Gateway (2021–2027), in prospective terms. In the concluding section, I summarize the content and introduce avenues for further research.

## The patterns of EU-CELAC cooperation in health and their multilateral engagement: a qualitative assessment of main initiatives between 2005 and 2019

The first interregional cooperation project with specific health concerns was “Strengthening the health sector in Latin America as a vector of social cohesion”, referred to as EUROsociAL/Salud, which was implemented between 2005 and 2009. In its website [22], we read that the contribution of health systems to social cohesion depends in large part on the equity of these systems in a broad sense. In this sense, health equity contemplates three dimensions: equity in the health status of individuals, access to services and treatments, and financing. The EUROsociAL programme assists with policies that address the first two.

EUROsociAL is multisector, being divided into five priorities that are part of the EU Cohesion Policy: administration of justice, education, taxation system, employment and health. The health sector, in turn, is divided into five areas: (i) development of social protection in health, (ii) good governance in health services, systems and hospitals, (iii) health services based on quality primary care and efficient and equal access to medication, (iv) public health policies and risk control, and (v) promotion of health policies in the community for the benefit of the most vulnerable and excluded sector [45]. The project is financed by the European Commission under the coordination of Spain (Fundación Internacional y para Iberoamérica de Administración y Políticas Públicas—FIIAPP), encompassing other European countries such as Italy, Germany and France. It also has two Latin American countries within the coordinating partners, which are Brazil (Escola Nacional de Saúde Pública Sergio Arouca—ENSP/Fiocruz) and Colombia (Agencia Presidencial de Cooperación Internacional de Colombia—APC), in addition to SICA (Sistema de la Integración Centroamericana).

As with other EU projects, EUROsociAL/Salud comes from the EU view that Latin America needs knowledge transfer to improve social cohesion and public policies. Therefore, these countries participate as receivers through a template of practices (inspections, workshops, internships, training activities, technical assistance), a timeline of exchanges, a set of goals to be achieved, and EU values that must be incorporated into mechanisms of social inclusion such as universal social protection, democratic participation, equality in the enjoyment of rights and access to opportunities. Although social cohesion was a main element of the EU-LAC Strategic Partnership initiated in 1999, we would need a better assessment of how such practices and exchanges took place in Peru, Panama and Uruguay, for instance.

The second project that can be considered part of the interregional cooperation in health is COPOLAD, the EU-CELAC Cooperation Programme on Drugs Policies, initiated in 2011 with EU funding [13]. Each phase has four years, and it is currently in its third phase, with a budget of €15 million from February 2021. It has nearly the same EU and LAC partners of EUROsociAL/Salud in addition to the EMCDDA (European Monitoring Centre for Drugs and Drug Addiction), with a focus on promoting technical cooperation based on scientific evidence as well as political dialog on drug policies between Latin America, the Caribbean and the EU. As regards to objectives, we read that they “will be fully respectful of the national sovereignty of each country and will be based on the demand raised by the participating countries themselves” (COPOLAD website).

I have already made a qualitative assessment of COPOLAD elsewhere [60] and will not discuss here the criticisms we could raise about how the EU communicates the programme, in light of how practices take place and how LAC countries understand the cooperation. After more than ten years since task forces were designated for implementation, expressions such as triangular cooperation, south‒south cooperation and national sovereignty began to emerge, at least in discourse, from the EU side.

The third project, and I would say the most specific in terms of health cooperation, was the “EU-LAC Health (2011–2017): Roadmap for Cooperative Health Research”. The five-year project is cofunded with the support of the European Community’s Seventh Framework Programme (FP7/2007–2013) and was presented on 29 May 2012 by its coordinator, Carlos Segovia, Deputy Director of International Research Programmes and Institutional Relations of the Institute of Health Carlos III (Spain), at the Open Information Day for the 7th call of FP7 Health Theme, as we can see in a press release of the event available online. Among the project partners, we have ISCIII and INNOVATEC (Spain), RIMAIS (Costa Rica), COHRED (Switzerland/Mexico), DLR (Germany), FIOCRUZ (Brazil), MINCyT (Argentina), and APRE (Italy).

According to its website [18], the EU-LAC Health is a project aimed at defining a Roadmap to support cooperative Health Research. A key aspect of the project will include linking and coordinating two important policy areas: science and technology policy (research) and international development cooperation. The EU-LAC Health is to be implemented through 6 different thematic areas: State of Play Analysis, Operational Road-mapping, Roadmap Consultation, Public Presentation, Final Dissemination and Management.

In November 2012, the project launched the first newsletter with main outcomes from the project activities, which, by that time, were basically an expert workshop held in Fiocruz (Brazil) and another one called ‘Scenario Building Workshop’ held in Buenos Aires, “in order to sort possibilities for a common funding of biregional research cooperation initiatives” and to prepare for the second one, to be held in Italy in 2013 [19]. Other newsletters were published over time, always indicating future activities.

We also have a kind of evaluation published in 2018: funded by the project and authored by researchers from Spain, Italy, Germany and Brazil who have participated in all activities of the project, already in the abstract we read that “EU-LAC Health represents a successful example of biregional collaboration and the emerging networks and expertise gathered during the lifetime of the project have the potential to tackle common health challenges affecting the quality of life of citizens from the two regions and beyond” ([41]:1). Although they were not independent actors but participants of the project, we can say that these are experts working in research and national health institutions. Among the main outcomes, the first is the EU-LAC Health Strategic Roadmap [17] which, according to the authors, “the methodology used for its definition is sound, the procedures have been tested, and the areas of common interest have been demonstrated to be of interest for R&I funding agencies and researchers. Those arguments make the roadmap a useful guide for policy-makers interested in biregional R&I collaboration” (op cit:7).

The Roadmap has seven sections: Context, Vision and Mission, Objectives and Principles, Swot Analysis, Scientific Research Agenda, Governance, and Roadmap Timeline 2015–2020. The authors detail what has been done in each of the six thematic areas mentioned above, relating to the main goals previously set. Other outputs cited in the publication were a network for collaboration among scientists, policy-makers and R&I funding agencies and the establishment of a coordinating body for future EU-LAC collaboration in health R&I.

### Multilateral engagement: EU-LAC support of the Oslo Group resolutions approved in the UN General Assembly (2008–2020)

The *Foreign Policy and Global Health Initiative* (FPGHI) was launched in NY in September 2006. In March 2007, the Ministers of Foreign Affairs of Brazil, France, Indonesia, Norway, Senegal, South Africa, and Thailand issued the "Oslo Ministerial Declaration—Global Health: a pressing foreign policy issue of our time" [27]. Since 2008, every year the Oslo Group, which is how the FPGHI became known, approves a resolution at the UN General Assembly (UNGA). After mapping the EU-LAC engagement, as sponsors and/or supporters, in each of the thirteen UNGA resolutions (until 2020),^6^ in addition to analyzing six Ministerial Communiqués, we have three resolutions that present the highest engagement among countries of both regions:

- 2009, A/RES/64/108, about reinforcing the interdependence between foreign policy and global health to coordinate efforts against the H1N1 pandemic throughout local, regional and global levels;
- 2010, A/RES/65/95, about considering Universal Health Coverage a central factor for the social determinants of health;
- 2012, A/RES/67/81, about financing mechanisms for enlarging systems of Universal Health Coverage (UHC).

The Oslo Declaration, by its turn, has an agenda organized around three main themes: ‘Capacity for global health security’; ‘Facing threats to global health security’; ‘Making globalization work for all’. The first theme has three specific actions: preparedness to respond to health risks and threats, control of infectious diseases, and strengthening human resources for health. The second theme has four specific actions, all related to conflicts, threats and natural disasters. The third theme has three specific actions, which are development, trade policies and measures to implement and monitor agreements, and improve governance for health. According to these findings regarding the UNGA resolutions, it is possible to say that the EU-LAC cooperation is potentially more effective within the scope of the third theme, which actions reflect the focus on development and trade.

### Results

For what we have seen until the COVID-19 pandemic, projects on interregional cooperation, such as EUROsociAL/Salud, COPOLAD and EU-LAC Health, approached different dimensions within a pattern of *development through health* that is part of a revisionist behavior adopted by both partners, although by different means, throughout the cooperation process. Revisionist behavior, as stated in the introduction, is likely to be seen in longstanding relations among actors with power asymmetry. It is therefore a behavior through which political coordination does not undermine respective interests, preferences, instruments and worldviews that may be different and non-negotiable. Social cohesion and health equity, technical assistance and political dialog on drug policies, and strengthening of health R&I collaboration are goals that represent the politics of cooperation, that is, the common denominators which encompass what each partner expected from the projects. Social development is indeed the premise of consensus-building between policymakers in both regions, through which they achieve significant outputs for global governance for health. In many regards, these projects reflect what is agreed upon in UNGA resolutions, especially in their social dimensions, such as the emphasis on social determinants of health and the enlargement of public systems of UHC.

Therefore, we can say that until the COVID-19 pandemic, the EU-CELAC health cooperation has characterized an approach of development through health within a two-way revisionist behavior embedded in those projects. And that, in practice, the projects were aligned with their multilateral engagement in the UN and in declarations for the occasion of EU-LAC summits over the period. Despite expected disagreements likely emerging out of their essential differences and asymmetries, both regions recognized potential issue areas in which a constructive dialog and policy-oriented outputs were reached. Foreign policy and multidimensional cooperation, embracing from local farmers to academics, have historically favored interregional governance for health. In the next section, I analyze whether and how this has changed since COVID-19.

### After the COVID-19 pandemic: any changes?

The first important move occurred after the pandemic came from the EU and, as we will see, affected the CELAC through some changes in the partnership itself. By the end of 2021, in a political response to the pandemic, the European Commission and the EU High Representative have set out the Global Gateway, a new European foreign policy strategy. As regards the budget, “between 2021 and 2027, Team Europe, meaning the EU institutions and EU Member States jointly, will mobilize up to €300 billion of investments for sustainable and high-quality projects, taking into account the needs of partner countries and ensuring lasting benefits for local communities”. It is also expected that the strategy will “create opportunities for the EU Member States’ private sector to invest and remain competitive, while ensuring the highest environmental and labor standards, as well as sound financial management” (Global Gateway website) [15].

Before exposing what is at the core of EU expectations for health, it is important to say more about the Team Europe approach, as it is the group responsible for allocating the budget and sizing the implementation of the Global Gateway strategy. In the website “Team Europe approach: leadership, cooperation, resources”, we find that Team Europe consists of the European Union, EU Member States — including their implementing agencies and public development banks — as well as the European Investment Bank (EIB) and the European Bank for Reconstruction and Development (EBRD). It offers a joint programming tracker with an overview on Team Europe Initiatives (TEIs) by country and region in which we see that thus far, for the LAC region, health is not yet contemplated (Team Europe Initiatives and Joint Programming Tracker website [29]) despite being one among the five key areas (digital sector, climate and energy, transport, health, education and research) selected under the Global Gateway for the EU-CELAC partnership from 2021.

Returning to EU expectations for health within the Global Gateway, we have a summary provided by the DG for International Partnerships in its website:

“Global Gateway will prioritize the security of pharmaceutical supply chains and the development of local manufacturing.^7^ (…) However, health issues extend beyond the pandemic. Thus, the Global Gateway will also facilitate investment in infrastructure and the regulatory environment for the local production of medicine and medical technologies. This will help integrate fragmented markets and promote research and cross-border innovation in healthcare, helping us to overcome diseases such as COVID-19, malaria, yellow fever, tuberculosis or HIV/AIDS” (DG for International Partnerships website).

In addition to this summary, we also have an [16], which starts by saying “The first two essential priorities are: investing in the well-being of all people and reaching universal health coverage with stronger health systems. The third core priority is combatting current and future health threats, which also requires a new focus. It calls for enhanced equity in the access to vaccines and other countermeasures,for a *One Health approach*,^8^which tackles the complex interconnection between humanity, climate, environment and animals” [16]:6). In the report, we have an agenda leading up to 2030 with three policy priorities—“2.1. Deliver better health and well-being of people across the life course; 2.2. Strengthen health systems and advance universal health coverage; 2.3. Prevent and combat health threats, including pandemics, applying a One Health approach -, provides for twenty guiding principles to shape global health, makes concrete lines of action that operationalize those principles, and creates a new monitoring framework to assess effectiveness and impact of EU policies and funding^9^” (op cit:8).

The EU understands itself as having a unique potential to drive international cooperation, expand partnerships and promote health sovereignty “for more resilience and open strategic autonomy supported by partners’ political commitment and responsibility” (op cit:6). Therefore, which kind of changes in EU-CELAC interregional cooperation in health pushed by the Global Gateway and EU Global Health Strategy 2022 can we expect? It seems that this is not an easy question and requires a careful analysis of the documents and speeches mobilized thus far. I propose some ideas in this regard: on the one hand, it is notable that the second policy priority (‘strengthen health systems and advance universal health coverage’)^10^ recovers the Oslo Group resolutions in which the EU and CELAC have reached more consensus and support, in addition to being in line with the two main joint programmes of the past, EUROsociAL/Salud and EU-LAC Health.

On the other hand, with regard to the third policy priority ("prevent and combat health threats, including pandemics, by applying a One Health approach"), it could be interpreted as the novelty promoted by the COVID-19 pandemic, although in reality, only the mention of the ‘One Health approach’ constitutes an innovation. This can be evidenced, for instance, in the 2009 UNGA resolution approved by the Oslo Group, in which the H1N1 pandemic was the target underpinning necessity to ‘coordinate efforts to prevent and combat health threats in local, regional and global levels’. In the same way, other diseases that are long-standing health threats dealt within the EU-CELAC interregional cooperation since at least 2005, such as malaria, yellow fever, tuberculosis and HIV/AIDS, are also mentioned in the Global Gateway for continuing cross-border research and innovation. Therefore, at least in terms of language, of the way ideas are presented and discourses are written, I do not see a stark turnaround. Even so, can we still expect change?

### Looking deeper at the EU Global Health Strategy

In the report, each policy priority is developed through guiding principles. When we zoom in on guiding principles of the second policy priority, we see what I just mentioned before: at least in the way they are stated, they remain aligned with the path of EU-CELAC interregional cooperation in health to date, characterized by a pattern of development through health. For this reason, I focus on the third policy priority to explore subsidies for us to reflect upon the following question: should we analyze this interregional partnership in health from 2022 onward in light of a revisionist behavior characterized by ‘reviewing previous disagreements’ or a reformist behavior identified by ‘setting new priorities’? To what extent could it be said that the pattern of cooperation has changed?

Having a closer look at the third priority, ‘Prevent and combat health threats, including pandemics, applying a One Health approach’, we find guiding principles 7 to 11:

- GP 7: Strengthen capacities for prevention, preparedness and response and early detection of health threats globally;
- GP 8: work toward a permanent global mechanism that fosters the development of and equitable access to vaccines and countermeasures for low- and middle-income countries;
- GP 9: negotiate an effective legally binding pandemic agreement with a One Health approach and strengthened International Health Regulations;
- GP 10: build a robust global collaborative surveillance network to better detect and act on pathogens;
- GP 11: apply a comprehensive One Health approach and intensify the fight against antimicrobial resistance.

To answer the above questions, I will base myself on these guiding principles and add what we have to date: since the EU published the report, three initiatives with CELAC have been announced by the Directorate-General for International Partnerships within the framework of the EU Global Gateway. They are:

1. 22 June 2022: “EU-Latin America and Caribbean Partnership on manufacturing vaccines, medicines and health technologies and strengthening health systems” [7];
2. 21 March 2023: “EU – Latin America and Caribbean high-level pharmaceutical forum to promote local manufacturing”;
3. 17 July 2023: “EU builds new partnership for improved Latin American and Caribbean health technologies with Pan American Health Organization” [8].

As we can see, all of them are placed within GP 7, which is part of seven lines of action. I reproduce such lines in the figure below.

Regarding the first initiative (‘EU-LAC Partnership on manufacturing vaccines, medicines and health technologies and strengthening health systems’), which seems to be the most robust, we read in the EU communication that it “will complement and further enhance social, economic and scientific ties between the two regions. It will boost Latin America's manufacturing capacity, foster equitable access to quality, effective, safe and affordable health products and help strengthen health resilience in the region to tackle endemic and emerging diseases, and enhance capacities to cope with noncommunicable diseases” (DG for International Partnerships website, News Communication section).

The second initiative (‘EU-LAC high-level pharmaceutical forum to promote local manufacturing’) is a development of the first. The Commissioner for International Partnerships Jutta Urpilainen and Commissioner for Internal Market Thierry Breton hosted in Brussels the EU-LAC High-level Forum *Sharing pharmaceutical innovations* under the Global Gateway [6]. Political leaders, technical experts, pharmaceutical companies, entrepreneurs, investors, and financing institutions from both regions were brought together to explore collaboration, for instance, in effective and affordable pharmaceutical innovations (DG for International Partnerships website, Conferences and Summits section).

The third initiative (‘EU builds new partnership for improved LAC health technologies with PAHO’) emerged from the EU-CELAC Summit held on 17 and 18 July 2023^11^ and was also a development of the first initiative. Ms. Urpilainen and Director of Health Systems and Services of the Pan American Health Organisation (PAHO), Dr James Fitzgerald signed a €3,8 million agreement building a partnership to strengthen LAC access to healthcare technology. The contribution agreement supports the main objectives of the EU-LAC partnership on health, launched by Ms. von der Leyen and Mr. Sánchez in June 2022 (first initiative listed). It focuses in particular on strengthening regulatory frameworks, technology transfers and increasing manufacturing capacities.^12^

After the Summit, an EU-CELAC Roadmap for 2023 to 2025 [14] indicated that a High-Level event on “Health Regulatory Frameworks” is planned for November 2023, and meetings on Health Self-sufficiency involving regulatory authorities from both regions are planned for 2024–2025. Finally, in the Declaration of the EU-CELAC Summit [4], we read on paragraph 30, page 8:

“We express our commitment to take forward the biregional partnership on local manufacturing of vaccines, medicines, and other health technologies, and strengthening health systems resilience to improve prevention, preparedness, and response to public health emergencies, in support of the CELAC Plan on Health Self-Sufficiency [see the link to access the Plan in footnote 6]. We look forward to the progress of the ongoing discussions on a new legally binding instrument on pandemic prevention, preparedness, and response in the framework of the World Health Organization, with the aim to agree it by May 2024”.

### Results

Taking these primary sources and empirical examples as references for our analysis, the research indicates that the kind of changes in EU-CELAC interregional cooperation in health reflects the dominance of EU interests. Considering the material produced from the EU Global Gateway strategy, launched after the COVID-19 pandemic, until the last EU-CELAC Summit, we can see that the main pattern of interregional cooperation moved from development through health to the current economy-driven health development – a movement clearly propelled by the EU by means of elevating health technologies and manufacturing as priorities despite knowing the enormous structural differences between both regions in this regard.

First and foremost, health technologies and manufacturing in CELAC are mainly conducted with public investment and in public institutions, while in the EU, this field is dominated by *big pharma*—private transnational companies, among the most profitable and richest in the world, that also count on EU subsidies (Polish Polpharma is a good example^13^) and normative facilities. However, in cooperation with CELAC, the centralization of the involvement of the private sector and the International Finance Corporation (IFC), as well as the harmonization of the economic interests in the health sector of several EU Member States, do not seem to be easy tasks for EU foreign policy to effectively implement this change of priorities declared in the official post pandemic documents and in the projects already underway.

With regard to the question of whether this political change on the EU side indicates revisionist or reformist behavior, i.e., a review of previous divergences or an attempt to establish new priorities, a qualitative evaluation of the primary sources from a historical perspective allows me to affirm that we are witnessing a reformist behavior on the part of the EU in its interregional cooperation with CELAC in the area of health. However, it is important to remember that, at the time of writing, the first initiatives have not yet been implemented, and therefore, we still have to wait to empirically evaluate the impacts of such change. We cannot anticipate reactions, contestations or resistance, but we do have lessons learned from the path of EU-LAC partnership in health issues that add valuable insights to our analysis, especially on how the historical differences in terms of health approaches between the two regions have been negotiated. In the next section, I give some of these insights, focusing on the issue of manufacturing pharmaceuticals and health technologies.

## Discussion

### Making sense of the EU-LAC Health Partnership within the EU Global Gateway (2021–2027)

To analyze potential points of disagreement at the implementation level of cooperation in manufacturing pharmaceuticals and health technologies, I take into consideration previous disputes involving the EU and CELAC countries located at the intersection between the regimes of health, trade and intellectual property rights (IPR). The EU-Brazil dispute over global access to medicines, which formally started in 2009 within the WTO, is illustrative. On the one hand, the EU focuses on the protection of patents within the IIPR regime and on combating counterfeit drugs, advocating that the defense of IPR and patent law are necessary conditions for investment in the research and technology of medicines conducted in developed countries, which guarantees global health by exporting ‘safe’ medicines worldwide. On the other hand, Brazil sees the EU drawing upon its bargaining power through trade and political leverage to validate its own regulation above multilateral ones at stake, such as the Doha Declaration on the TRIPS Agreement and Public Health (2001), hampering the transit of generics.

Under the EU Regulation 1383/2003 and in response to complaints of patent rights owners, Dutch customs authorities systematically confiscated in transit medicines between 2008–2009 at the Rotterdam port and Schiphol airport in Amsterdam, mainly from India to Africa and Latin America [46]:25, where the author gives a complete explanation about drug confiscations in European routes), alleging counterfeit and the violation of IPR contained in the WTO TRIPS Agreement (Trade-related Aspects of Intellectual Property Rights, 1994). For the first time, in early December 2008, Brazil and the EU reached the peak of their divergent perspectives about the right to health *vs.* IPR regulated by TRIPS. The dispute broke out: on 3 February 2009, the Permanent Representative of Brazil to the WTO Ambassador Roberto Azevedo made an intervention at the WTO General Council (GC) on the seizure by Dutch authorities of a cargo of 570 kilos of losartan potassium docked in Rotterdam while in transit from India to Brazil [24].

Ambassador Azevedo set one leading point of Brazilian argumentation: the distinction between generics and counterfeit (“*The concept of generic must not be mistaken with counterfeit or pirated. Generic medicines are not substandard or illegal”*), reaffirming the irrelevance of patent law in the Netherlands, which was only the country of transit, supported by the principle of territoriality that is at the basis of the IPR regime (*“Whether or not the medicines were generic under the law of the country of transit is an irrelevant question”*). The dispute lasted until 2016, when the EC (Taxation and Customs Union) published a Commission notice in its website [10], informing that a new regulation concerning customs enforcement of IPR has replaced the EU Regulation 1383/2003, addressing the “specific concerns raised by India and Brazil on medicines in genuine transit through the EU which are covered by a patent right in the EU”. In an interview given in 2017, Celso Amorim, Minister of Foreign Affairs of Brazil from 2003–2010, summarized the dispute: “We have a very important IP system, one of the most developed IP institutes in the developing world, which gives expertise to other countries. So no, we’re not against IP at all. However, we have to see that life is above profit, and health is above patents” [25].

This is just one illustrative case, among other disputes between the EU and counterparties such as India, African and LAC countries that are well documented in different UN stages, such as the Intergovernmental Working Group for establishing a legally binding agreement on Business and Human Rights settled within the Human Rights Council in 2014 and the Dispute Settlement Body of the World Trade Organization [32, 59]. The EU has consistently acted in the best interest of its pharma companies, and this behavior has been denounced by international NGOs such as Health Action International (2018, [23]) and Oxfam, as we can see in a press release published in April 2023 on its website under the title “EU pharma legislation ‘total hypocrisy’ while undermining health in poorer countries, campaign says” [28]. In addition, those NGOs and other researchers from the South [34, 40, 43, 44] have been systematically reporting that EU trade agreements contain TRIPS + , which are basically additional measures to strengthen IPR also upon health products in detriment of health safeguards regulated by the Doha Declaration of 2001 (see the Doctors Without Borders Access Campaign to further information on EU´s use of TRIPS + in its trade agreements) [9]. Recently, we have witnessed this concern in regard to the EU-Mercosur FTA [38].

**Fig. 1:**
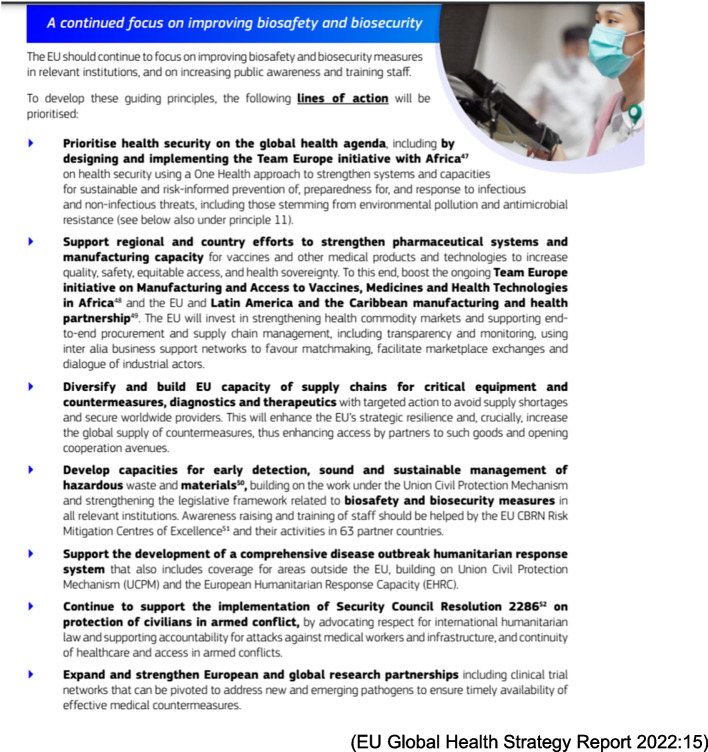
Lines of action driving also guiding principle 7

The second line of action of guiding principle 7 in the [16], as stated in Fig. 1, is worth recalling:

“Support regional and country efforts to strengthen pharmaceutical systems and manufacturing capacity for vaccines and other medical products and technologies to increase quality, safety, equitable access, and health sovereignty. To this end, boost the ongoing Team Europe initiative on Manufacturing and Access to Vaccines, Medicines and Health Technologies in Africa and the EU and Latin America and the Caribbean manufacturing and health partnership. The EU will invest in strengthening health commodity markets and supporting end-to-end procurement and supply chain management, including transparency and monitoring, using inter alia business support networks to favor matchmaking, facilitate marketplace exchanges and dialog of industrial actors” (emphasis in the original).

In the history of EU relations with LAC countries, it is no exaggeration to say that disputes involving health, trade and IPR have been the hardest ones, very much due to the lobby of private pharmaceutical European industries – who are exactly the private actors that will be called upon to take part in manufacturing and health partnerships with CELAC. In other words, the above line of action of guiding principle 7 goes in a direction *opposed* to the one pharmaceutical lobby—mainly represented by the European Federation of Pharmaceutical Industries and Associations—is used to expect and to get from the EU.

Therefore, the crucial question is ‘how will the EU operationalize the support of regional and country efforts in LAC to strengthen their pharmaceutical systems and manufacturing capacity for vaccines and other medical products and technologies *without* harnessing the European pharma sector´s profit and interests—which is a key sector for being the major contributor to the EU economy, as recognized in the *2020 Pharmaceutical Strategy for Europe*?’ Another doubt is how the EU would support end-to-end procurement and supply chain management, including transparency and monitoring, without its pharma sector´s full cooperation? These are still open questions that must be seen empirically over the process of implementation.

## Conclusion

The fact that Latin America has been a global player in health since the XIX century, having participated in the constitution of norms, organizations, practices and approaches as much as other countries from the North did [42, 47], had compelled the EU to tailor cooperation toward social cohesion and research and innovation (R&I), as we have seen in the EU-funded projects implemented until 2019. Nonetheless, the history of implementation of common projects shows that they were successful *despite* disagreements in how both regions understand health. In the first section, I presented the pattern that guided EU-CELAC cooperation in the field of health until the COVID-19 pandemic—which I call *development through* health—with a view to our objective of identifying patterns of practices and what/how they have changed.

This line of reasoning can lead to further research toward positive implications for the EU’s regional health partnerships more broadly. By opening up the projects, analyzing interactions and revealing how mechanisms for accommodation and negotiation of disagreements were mobilized, this kind of research would have the potential to enhance our knowledge about how revisionist behavior is practiced in asymmetric partnerships, such as the one between the EU and sub-Saharan Africa within the European and Developing Countries Clinical Trials Partnership (EDCTP). Because each project has its own historiography, it is valuable for researchers to refine the meaning of the ‘politics of cooperation’ by uncovering how other partners construct health cooperation with the EU.

For instance, Berner-Rodoreda et.al. explain that, until 2010, in the EU Communication on its role in Global Health, the aspect of protection against threats was “led to the question of whom health security is for or even what it should entail or how ‘security’ should be defined and specified” (2019:2). Securitization of health is an approach that has been increasingly nested in the EU [33] and, because the EU is commonly afforded more financial resources in the scope of regional partnerships, its approaches and meanings on central aspects that drive cooperation are unavoidably relevant. Nonetheless, that fact does not say much about how partners react, adapt, reject, contest and interact with EU counterparts at the negotiation and implementation levels. To assess this kind of knowledge is what I meant by empirically refining our understanding about the politics of cooperation as a contribution to the literature on EU´s regional health partnerships.

In section two, I concentrated on a detailed analysis of key EU documents to understand what was in there beyond the surface of vague statements that usually announces policy priorities for a broad audience. We saw that even after the pandemic, the language used by the EU in these priorities resembled resolutions of the Oslo Group, as they represent consensus-building within the UNGA about foreign policy and global health. Therefore, looking deeper at the EU Global Health Strategy 2022, I claim that EU reformist behavior is set in specific guiding principles and lines of action of the third policy priority. This means that by prioritizing different principles and policy instruments^14^ that came up from an internal revision of its own foreign policy direction after the COVID-19 pandemic, the EU did not previously negotiate what/ how should be changed in its health cooperation with CELAC. The outcome is that, this time, the changing pattern of such cooperation reflects the dominance of EU interests – a change from development through health to *economy-driven health development* – probably influenced by the geopolitical context that made China a relevant health partner for CELAC during the pandemic.

By an economy-driven health development, I mean the choice for a pattern of cooperation focused on strengthening the development of specific health technologies for intervention in people's bodies, which depends foremost on investment and financing of innovation, i.e., the strengthening of the IPR regime. In turn, the IPR regime is a historical node in EU-CELAC relations, mostly with regard to the health industry. Because of, in practice, it remains to be seen how the three initiatives with CELAC already announced by the DG for International Partnerships under the EU Global Gateway between June 2022 and July 2023 will be implemented, in section three, I recover some previous lessons and insights about disputes involving the EU and CELAC countries located at the intersection between health, trade and IPR regimes. I also include the voice of international NGOs such as Doctors Without Borders and Oxfam, as well as of civil society organizations from the South. Alongside them, there are a number of research articles and reports propagated by European civil society organizations manifesting against the EU preference for trade when health is at stake [2, 30, 50, 58, 63].

The goal here was to demonstrate how hard disputes involving health, trade and IPR are precisely because of the lobby, interests and profit of European pharmaceuticals, whose collaboration is, from now on, essential for the success of EU-CELAC health cooperation. Moreover, being one of the main contributors to the EU economy, the pharma sector enjoys subsidies, incentives and normative protection at the supranational level, represented by the European Commission, its related DGs and complementary agencies. Since cooperation implies sharing knowledge and expertise to strengthen pharmaceutical systems and manufacturing capacity for vaccines and other medical products and technologies in CELAC countries, further research is necessary to follow, first, how the EU will manage to obtain private pharma sector collaboration. This is a crucial aspect with implications perhaps also to its global health strategy which, besides the Global Gateway, encompasses for instance the Horizon Europe R&I Framework initiated in June 2018 [21], in which “European industrial interests were found to dominate with regard to Global Health innovations” [35].

Despite this scenario, it has become clear that the players in both regions have learned to adapt to each other without major losses for their populations, who are presumably the main beneficiaries of cooperation. We therefore have reason to believe that they will continue to draw their own preferences from the partnership. CELAC would have much to gain in centralizing the engagement of PAHO and ECLAC on the basis of the “Regional Plan for Self-sufficiency in Health Matters”, for instance, in joint activities with the European private sector. In addition, in doing so, they already know where main disagreements are to be expected. It remains to be seen the power of an economy-driven health agenda as the new pattern to enforce EU priorities in the context of health cooperation with CELAC – this is the main factor that will define how (and not just which) competing interests and capacities will be accommodated.

In this regard, the WHO Commission on Social Determinants of Health (SDH) states that “Together, the structural determinants and conditions of daily life constitute the social determinants of health and are responsible for a major part of health inequities between and within countries” ([3]:1). SDH offers a framework *through which* other common goals such as economic growth and security are achieved and entails three principles for action: “Improve the conditions of daily life”, “Tackle the inequitable distribution of power, money, and resources” and “Measure and understand the problem”. These principles might not drive the aim but rather the necessary results of global governance for health. Can the EU-CELAC health partnership, after the pandemic, continue to contribute to these outcomes by emphasizing equitable access and health sovereignty, negotiating disagreements over the means to reduce asymmetries and maintaining the will to achieve the respective priorities through cooperation and multilateralism? This is the most significant question motivating researchers and societies to observe future developments.

## Data Availability

All data generated or analyzed during this study are included in this published article.

## Footnotes

1 For more information about EU´s other regional health partnerships see, for instance, the website of Horizon Europe where there is a list of partnerships in the area of health, beginning with the one with sub-Saharan Africa. Available in: https://research-and-innovation.ec.europa.eu/funding/funding-opportunities/funding-programmes-and-open-calls/horizon-europe/european-partnerships-horizon-europe/health_en.

2 Such as the Commission´s strategy “Together for Health” (2008–2013) and the *EU Global Health Strategy* that will be scrutinized in subsequent sections of this article.

3 Such as the European Medicines Agency (EMA), the European Center for Disease Prevention and Control (ECDC), the European Health and Digital Executive Agency (HaDEA) [20].

4 The official name of the Oslo Group is “Foreign Policy and Global Health Initiative”. It was launched in New York in September 2006. On March 2007, the Ministers of Foreign Affairs of Brazil, France, Indonesia, Norway, Senegal, South Africa and Thailand issued the "Oslo Ministerial Declaration—Global Health: a pressing foreign policy issue of our time”. Since then, every year the Oslo Group has a resolution approved at the United Nations General Assembly.

5 Ministerial Meeting "Latin American and Caribbean access to the experimental vaccine against COVID-19"; Videoconference "Accelerating access to COVID-19 vaccines in Latin America and the Caribbean"; Invitation to join the Coalition for Epidemic Preparedness and Innovation (CEPI); Virtual Ministerial Meeting on Health Affairs for the Attention and Monitoring of the COVID-19 pandemic in Latin America and the Caribbean; Social Communication Strategy on CELAC Social Networks; Special Communiqué on the timely monitoring of the outbreaks of the new coronavirus; Establishment of the Network of Specialists in Infectious Agents and Emerging and Reemerging Diseases. I am thankful to reviewer 2 for listing these initiatives.

6 UN General Assembly Resolutions Tables, available in: https://research.un.org/en/docs/ga/quick/regular/78

7 Alongside this strategy, another one that stands out is the *2020 Pharmaceutical Strategy for Europe*, which defines the EU financial contribution, co-investment from partners and pooling with other international actors [1].

8 A full definition of One Health is provided by OHHLEP under Tripartite and UNEP support OHHLEP's definition of "One Health" (who.int)—The Food and Agriculture Organization of the United Nations (FAO), the World Organization for Animal Health (OIE), the United Nations Environment Programme (UNEP) and the World Health Organization (WHO) welcome the newly formed operational definition of One Health from their advisory panel, the One Health High Level Expert Panel (OHHLEP), whose members represent a broad range of disciplines in science and policy-related sectors relevant to One Health from around the world. The four organizations are working together to mainstream One Health so that they are better prepared to prevent, predict, detect, and respond to global health threats and promote sustainable development [26].

9 This strategy should be read together with the first State of Health Preparedness Report, available in: https://health.ec.europa.eu/publications/state-health-preparedness-report_en.

10 On page eleven of the report we have a detailed description of the second priority, which is developed through guiding principles 3 to 6.

11 To better grasp the EU rationale for the EU-CELAC Summit, see a Joint Communication to the European Parliament and the Council entitled “A New Agenda for Relations between the EU and Latin America and the Caribbean”, which has been issued on 7 June 2023, specially pages 12-14. Available in: https://ec.europa.eu/commission/presscorner/detail/en/ip_23_3045 [11]

12 The EU affirms that the focuses are fully aligned with the “Regional Plan for Self-sufficiency in Health Matters”, endorsed by CELAC in September 2021, available in: https://www.cepal.org/en/publications/47253-plan-self-sufficiency-health-matters-latin-america-and-caribbean-lines-action-and.

13 See “Polpharma seeks EU funding to conduct R&D projects”, available in: https://polpharma.pl/en/eu-funding/

14 Different principles and policy instruments such as the guiding principles 7 “strengthen capacities for prevention, preparedness and response and early detection of health threats globally” and 8 “work toward a permanent global mechanism that fosters the development of and equitable access to vaccines and countermeasures for low- and middle-income countries”, as well as the policy instrument “Team Europe initiative on manufacturing and access to vaccines and health technologies in Africa and the EU and LAC manufacturing and health partnership” stated in the second line of action, in Fig. 1.
